# A novel single nucleotide polymorphism in exon 3 of *MYOC* enhances the risk of glaucoma

**DOI:** 10.1371/journal.pone.0195157

**Published:** 2018-04-09

**Authors:** Sabeen Nazir, Maryam Mukhtar, Maryam Shahnawaz, Shaima Farooqi, Naz Fatima, Rabia Mehmood, Nadeem Sheikh

**Affiliations:** 1 Department of Zoology, Quaid-e-Azam Campus, University of the Punjab, Lahore, Pakistan; 2 Department of Biomedical Engineering Center, Kala Shah Kaku Campus, University of Engineering and Technology, Lahore, Pakistan; Oregon Health and Science University, UNITED STATES

## Abstract

Genetic polymorphismsof *MYOC*alter the myocilin protein,which leads to disruption of thenormal regulation of intraocular pressure (IOP) that ultimately causes glaucoma.Theaim of the present study was to identify the polymorphism in exon 3 of the *MYOC* gene of theglaucoma patients in Lahore, Pakistan. We conducted a case-control study with 100 patients and 100 controls subjects. We extracted DNA from blood samples,amplified the target DNA fragmentby PCR, and identifiedpolymorphisms through sequencing. We observed that the allelic and genotypic frequencies of rs74315341 and rs879255525 were associated with glaucoma in our patient population. The polymorphism atrs74315341 led to the substitutionof serine for arginine,whereas the polymorphism at rs879255525 led to the substitution ofasparagine for lysine. The haplotype TGAAGCCATTTC was associated with disease onset, whereas the haplotype GGAAGCCATTTC was protective against disease development. In conclusion, weidentified *MYOC* gene polymorphisms in susceptible regions that were associated withglaucoma onset among the Lahore patient population.This is the first report to identify a novel mutation in rs879255525 in exon 3 of the *MYOC* genethat is associated withglaucoma.

## Introduction

Glaucoma is aprogressive optic neuropathy that leads to visual fieldimpairment[1]. Optic neuropathy is caused by rimming up or retrogression of the optic nerve,whichprompts the loss of fringe vision;if not cured,it can lead to irreversible visual impairment [2]. Primary open angle glaucoma (POAG) is a complex disorder with a major heritable component. The candidate genes associated with POAG onset are myocilin (*MYOC*); WD repeat domain 36; optineurin;cytochrome P450 family 1, subtype B,polypeptide 1; ankyrin repeat and SOCS-box containing 10and neurotrophin 4[3,4]. The first locus associated withPOAG was positioned inchromosome 1;the GLC1A, now known as *MYOC*, locusencodes the protein myocilin. Disease-related myocilin mutations are commonly foundin juvenile or early adult patientswith very high levels of IOP[5].

*MYOC*has3exons;most mutations have beenfound in the third exon, which encodes the olfactomedin-like domain [5].Myocilin forms part of themain structure of the eye,the trabecular meshwork, which regulates IOP[6–8].Mutations that change the structure of myocilin disrupts the normal regulation of IOP. Disease-related forms of myocilin undergo alteredprotein trafficking, leading to intracellular aggregation of the misfolded protein. The inability to properly release the protein enhances the IOP[9].

Genetic diseasesare increasinglyprevalentin Pakistandue to its relativelygeneticallyheterogeneous population. Common consanguineous marriageresults in frequent transmission ofmutationsthrough the generations. The glaucoma incidence rate in Pakistan is3.9% [10],but the exact genetic cause of this disease remains a mystery because of the unavailability of baseline data. Therefore,we designed a case-control study with theaim to determine the polymorphismsinexon 3 of *MYOC*in Lahore glaucoma patients.

## Materials and methods

### Sampling

The study was ethically approved by the Board of Studies of the University of the Punjab, Lahore. Sampling was carried out at Layton Rahmatullah Benevolent Trust, Lahore. After we obtainedwritten, informed consent from the patients or their guardians on the prescribed forms, we collected blood samples (3 ml) from 100 glaucoma patients and 100 healthy individuals in EDTA-coated tubes;we recorded the clinical characteristics of the subjectson performa. The inclusion and exclusion criteria for patient selection include IOP (tonometry), optic nerve damage (ophthalmoscopy), complete field of vision (perimetry), angle where the iris meets the cornea (gonioscopy), and thickness of the cornea (pachymetry).

### Genotyping

We extracted genomic DNA from each blood sample using the modified organic method [11]; we quantified the DNAusing a NanoDrop™ spectrophotometer. We amplifiedthe target sequence using previously reported primers [12].

We optimized the primers by gradient PCR and amplified the targeted sequence of 960 bp in 25-μl PCR mixtures containing 3 μl DNA template, 4 μl MgCl_2_(25 mM), 4 μl 10× PCR buffer, 3 μl dNTP mix (2.0 mM), 1.5 μl forward and reverse primers (10 pM), 0.5 μl Taq Polymerase (500 U; Thermo Fisher Scientific), and 7.5 μl DEPC water. The PCR cycle included an initial denaturation at 95°C for 5 min, followed by 30 cycles of 30s of denaturation at 95°C, 45s of annealing at 67.5°C, and 45s of extension at 72°C. This was followed by final extension at 72°C for 10 min.We sent the PCR products to Advance Biosciences International for sequencing.

### Sequence and statistical analysis

We visualized the sequences with BioEdit software and analyzed them usingthe Basic Local Alignment Search Toolfromthe National Center for Biotechnology(NCBI) and the University of California, Santa Cruz Genome Browser to identifysingle nucleotide polymorphisms (SNPs). All SNPs were assessed forHardy–Weinberg Equilibrium (HWE). We calculated the allelic and genetic frequencies and determined the associationof the *MYOC* gene polymorphisms with disease onset with thechi-squaredtest and Fisher’s test. We determined the linkage disequilibrium (LD) and performed haplotype analysis online with SHEsis software (http://shesisplus.bio-x.cn/SHEsis.html). We evaluated changes in amino acid sequence using MEGA 6 software.

## Results

Among the 100 patients, 40 males and 55 females had positive family histories of glaucoma, whereas none ofthe control subjectshad a positive family history. The mean age at the time of glaucoma diagnosis was 47.3 years formalesand52.5 years for females. The mean age of inclusion of diseaseformale and female patients was 51.6 years and 54.5 years, respectively.We identifiedrs74315341 by genotyping. rs74315341 comprisesthe replacement of guaninewiththymidine. Wealso identified a novel SNP that comprises the replacement of guaninewiththymidine. We submitted the sequence to the ClinVar NCBI database and theSNP was assignedthe novel number rs879255525. BothSNPs were inHWE (p>0.05). The allelic and genotypic frequencies of the SNPsare presented in Tables 1 and 2, respectively.The allelic and genotypic frequencies of rs74315341 and the novel SNP rs879255525 variedsignificantlybetweenthe patients and controls, and were significantly (*p*<0.01) associated with glaucoma onset.The change in the nucleotide sequence ofrs74315341 resulted in the substitution ofserine for arginineand the change in rs879255525 resulted in the substitution ofasparagine for lysine. The SNPs rs74315335, rs121909193, rs74315334, rs74315329, rs74315330, rs74315336, rs74315338, rs74315328, rs74315331, and rs74315332 wereassociated with glaucoma onset in our population, but the associations did not reach statistical significance.

**Table 1:** Allelic frequency distribution.

SNP number	Minor allele	Minor allele frequency		Major allele	Major allele frequency		Odds ratio	*p*-value
		Case	Control		Case	Control		
rs879255525	T	0.665	0.000	G	0.335	1.000	199.250946	0.016
rs74315341	T	0.660	0.000	G	0.340	1.000	197.014923	0.04

**Table 2:** Genetic frequency distribution.

SNP number	Genotype	Frequency(case/control)	*p*-value
rs879255525	GG	0.300/1.000	0.001
	GT	0.070/0.000	
	TT	0.630/0.000	
rs74315341	GG	0.210/1.000	0.015
	GT	0.260/0.000	
	TT	0.530/0.000	

Haplotype analysis indicated that the sequencesGTAAGCCTTTC and TGAAGCCATTTC appeared at higher frequenciesin patients than in controls, and that TGAAGCCATTTC was strongly associated with the onset of glaucoma (p = 0.005). On the other hand, GGAAGCCATTTC appeared athigher frequency in the controlsthan in the patients,indicating that itexerted a protective role against glaucoma onset.

**Fig 1:**
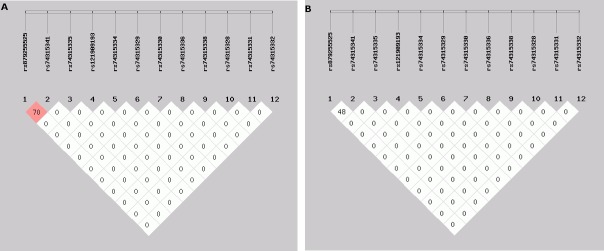
(a,b). Location and map of LDofSNPs onchromosome 1. The SNP numbers are represented at the top of the haploview. The pair-wise LD coefficient (r^2^) is presented at the top, and LD^*^ = D’.

The LD value for rs74315341 and rs879255525 was 0.703, suggesting that they are significant risk factorsforglaucomadevelopment.We did not observe significant LD between the SNPs, with the exception of rs879255525(Fig 1A and 1B).

## Discussion

More than 60 million people have been diagnosed withglaucoma,a complex group of optic neuropathies [13]. Mutations of the *MYOC* geneare associated with POAG onset in Chinese, French, Spanish, American, Australian, Canadian, Indian, Swiss, and Japanese populations[14–16].

We demonstrated that mutations in exon 3 of myocilinat the rs74315341 andrs879255525 polymorphic sites were associated with glaucoma onset in a Lahore patient group, which was consistent withthe role of rs74315341 in POAG developmentin an Australasian population [17]. Studies in Caucasian and Brazilian populationsalso found a significant association of this SNP with glaucoma [18,19].

We found that rs74315335, rs121909193, rs74315334, rs74315329, rs74315330, rs74315336, rs74315338, rs74315331, and 74315332 were not significantly associated with glaucoma onset in our patientpopulation. However, a previous a study reported that rs74315335 was significantly associated with POAG [20]. Similarly, rs74315334, rs74315329, and rs74315330 are significantly associated with glaucoma onset in an Australasian population [17].rs74315329 is a risk factor for disease onset in a Tasmanian population [21]. Furthermore, rs74315336 is significantly associated with hereditary glaucoma onset in the United States[22]and rs74315328 and rs74315331 have also been associated with glaucoma onset[23].

In the present study,we observed that theSNPschangedtheamino acid sequences and would, ultimately, alter the myocilin protein structure. Consistent with our results, previous studies havereported that the mutated myocilin protein becomesentangled in the cell in its altered forms [24,25].Heterodimers and heteromultimers with wild-type myocilinformwith altered myocilin proteins [26]. Large proteins aggregate in the endoplasmic reticulum as aconsequence of misfolded, disease-causing myocilin mutants. Altered myocilin secretionis alsosensitive to temperature, in support of the hypothesis that myocilin-induced glaucoma is a proteinconformational disease [27,28].

## Conclusions

Thus, polymorphismsin exon 3 of *MYOC*at the rs74315341 and rs879255525 polymorphic sitesare significantly associated with POAG onset in a Pakistani population.A large-scale survey should be conducted to evaluate the genetic factors associated with POAG to facilitate the identification and treatment ofsusceptible communities.
